# Supplementary material for: Combination of Mesenchymal Stem Cell-Delivered Oncolytic Virus with Prodrug Activation Increases Efficacy and Safety of Colorectal Cancer Therapy
Source: Biomedicines. 2021 May 13;9(5):548. doi: 10.3390/biomedicines9050548 (PMC8153168; doi:10.3390/biomedicines9050548)
Supplement: Supplementary file 1 [file biomedicines-09-00548-s001.zip › biomedicines-1205804-supplementary.pdf]

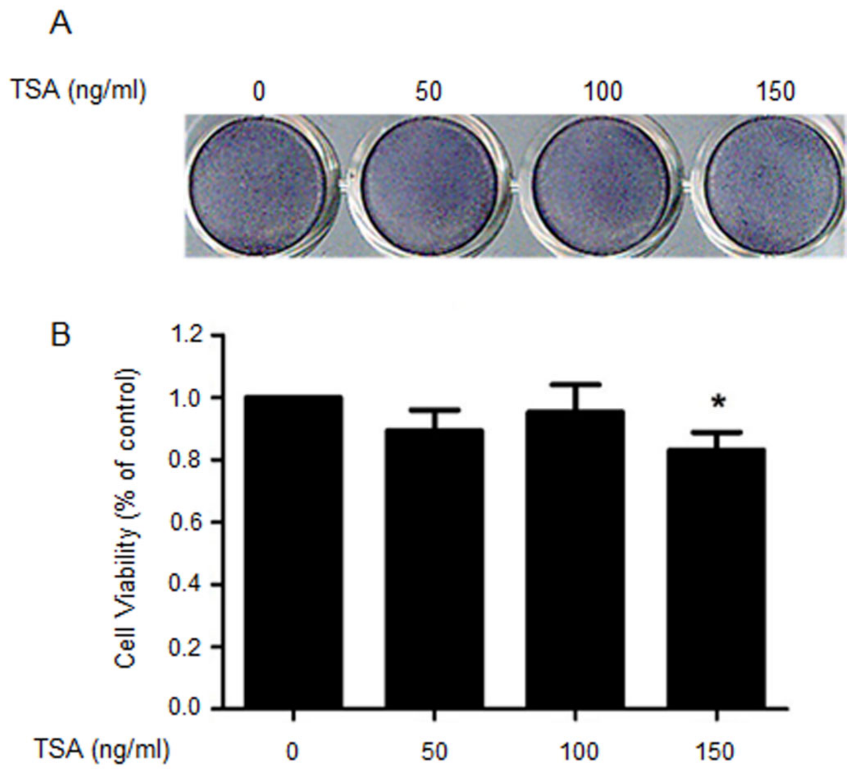

Figure S1. Identification of the optimal concentration of TSA. MSCs were treated with TSA at the dose of 50, 100 and 150 ng/ml for 24 h. Cell viability was determined by the crystal violate staining (A) and MTT assay (B). The results are presented as mean ± SD of three independent experiments. Asterisk indicates a significant difference (*P < 0.05 versus MSCs untreated with TSA).

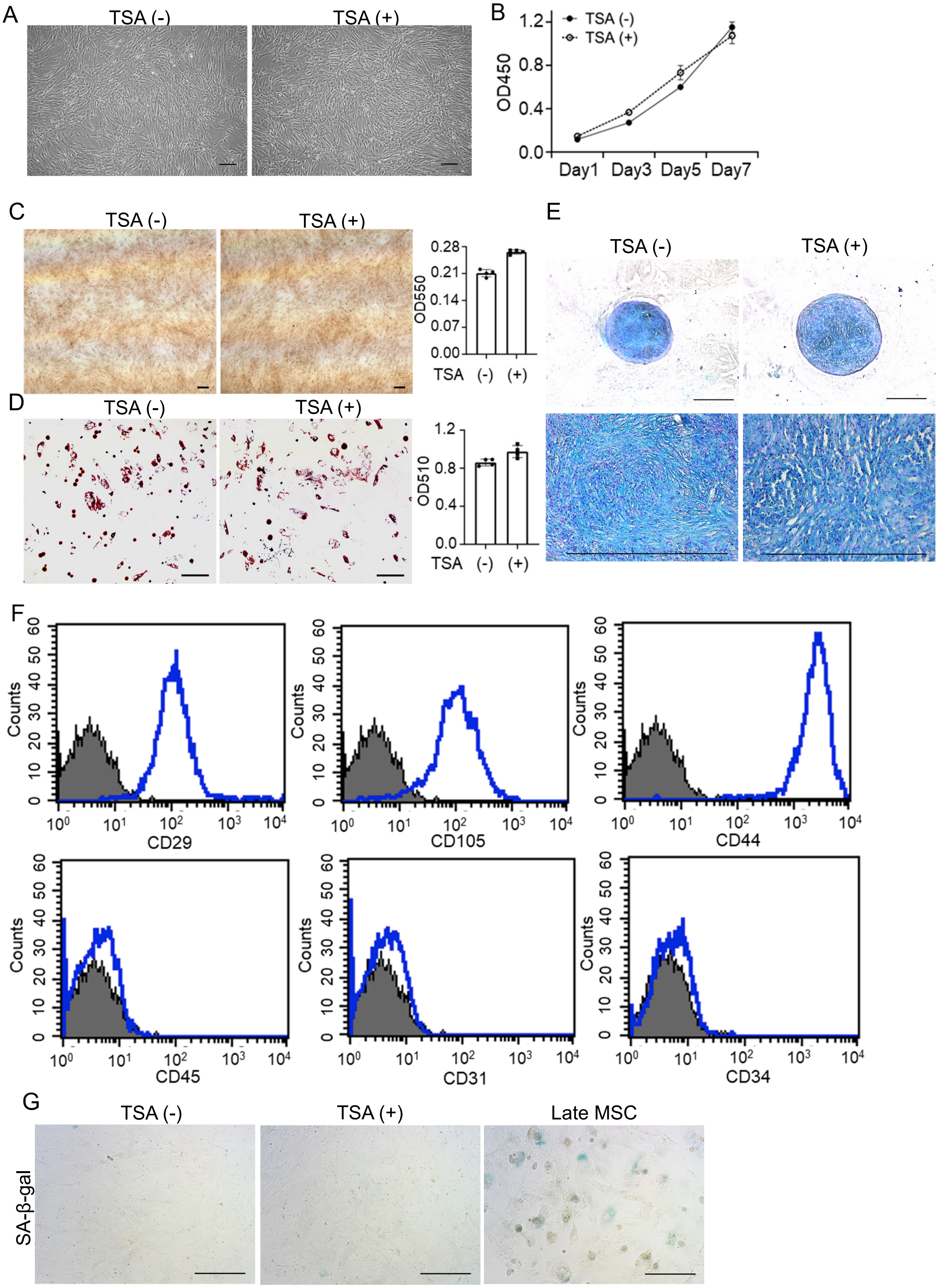
A
TSA (-)
TSA (+)
B
TSA (-)
TSA (+)
OD450
1.2
0.8
0.4
0.0
Day1
Day3
Day5
Day7
C
TSA (-)
TSA (+)
OD550
0.28
0.21
0.14
0.07
0.00
TSA (-) (+)
D
TSA (-)
TSA (+)
OD510
1.2
0.8
0.4
0.0
TSA (-) (+)
E
TSA (-)
TSA (+)
F
Counts
CD29
CD105
CD44
CD45
CD31
CD34
G
TSA (-)
TSA (+)
Late MSC
SA-β-gal

Figure S2. Characterization of MSC after TSA treatment. MSCs treated without or with 100 ng/ml TSA for 24 h were not different in (A) morphology, (B) proliferation capacity (WST1 assay), and differentiation potentials into (C) osteoblasts, (D) adipocytes, and (E) chondrocytes. (C Left) MSCs were induced for osteogenic differentiation and stained with Alizarin Red S at day 14. (C Right) Quantification of osteogenesis. (D Left) MSCs were induced for osteogenic differentiation and stained with Oil Red O at day 14. (D Right) Quantification of adipogenesis. (E) Pellets of MSCs were induced for chondrogenesis and stained with Alcian blue staining at day 21. (F) Flow cytometry for surface profile of TSA-primed MSCs. (G) SA-β-gal staining of senescent cells. The late passage of MSC was set as a control to show the reliability of staining. Scale bar, 200 μm.

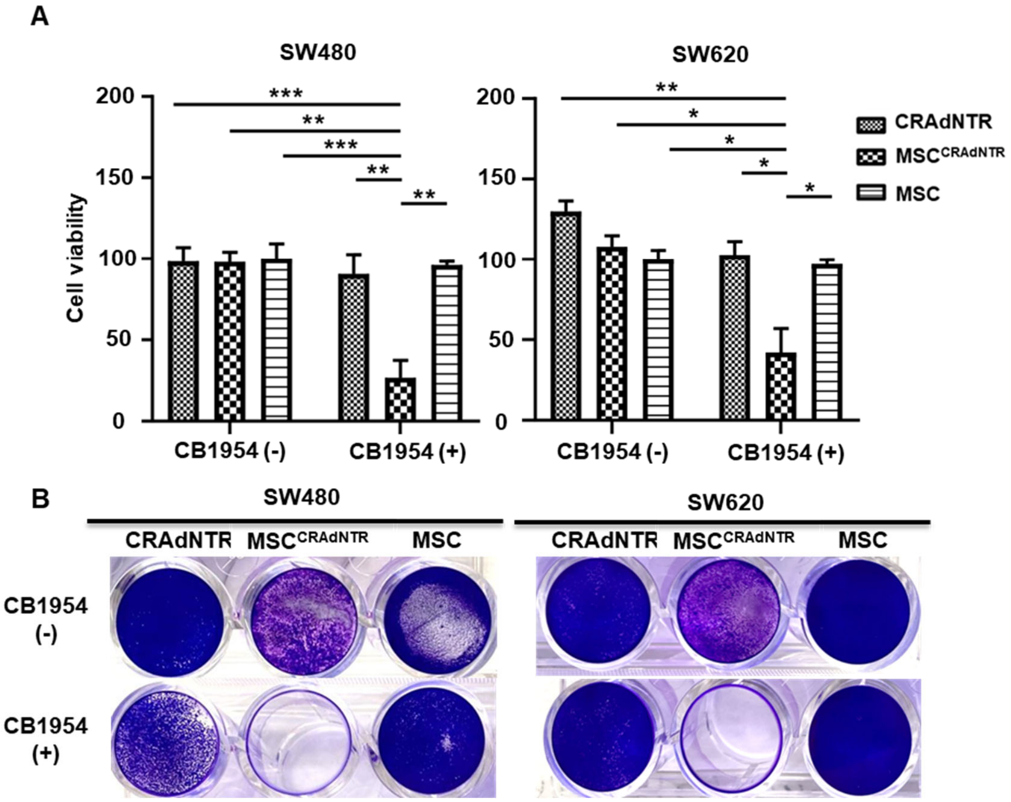

Figure S3. Combination of MSCs-delivered CRAdNTR with prodrug induces cytotoxicity in SW480 and SW620 in the presence of NAb in vitro. CRAdNTR, MSCs pretreated with 100 ng/ml TSA for 24h, followed by loading with CRAdNTR (MSC CRAdNTR), or MSCs pretreated with TSA alone (MSC) were seeded into wells pre-seeded with SW480 and SW620. The cell cultures were treated with anti-Adv neutralization antibodies (NAb) and CB1954 at indicated time shown in Fig. 3A. Aliquot of cells were subjected to (A) MTT assays, (B) crystal violet staining. Asterisk indicates a significant difference as determined by One Way ANOVA (****$p<0.0001$ versus other groups).

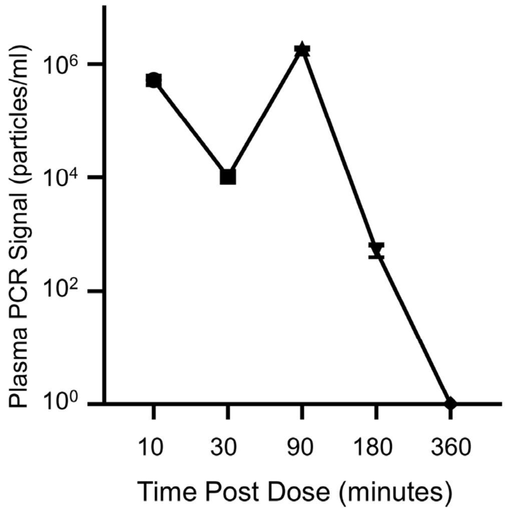

Figure S4. Pharmacokinetics and clearance of CRAdNTR. Tail vein blood samples of mice infused with 2×10^8 CRAdNTR viral particles were harvested at 10, 30, 90, 180, and 360 min post infusion for pharmacokinetic studies. The genomes of virus per 50 μl of blood were determined by quantitative-PCR.

Table S1. Primer sequences for real-time PCR and traditional PCR

Primer name	Sequence	Product size (bp)
**Real-time PCR**		
CAR	Forward 5’-AAATTTACGCTTAGTCCCGAAGAC-3’<br>Reverse 5’-CTGATATGTGCCAATATCTGACAG-3’	234
CXCR4	Forward 5’-CCTTATCCTGCCTGGTATTGTC-3’<br>Reverse 5’-CACCCTTGCTTGATGATTTCC-3’	201
GAPDH	Forward 5’-CTTCGATGATGCCGCAGTG-3’<br>Reverse 5’-GGGCTCAGGTACTCCGAGG-3’	675
E1A	Forward 5’-CCGACACCGGGACTGAAAAT-3’<br>Reverse 5’-AGCTGGTCCAAAAGACTGGC-3’	86
**Traditional PCR**		
E1A	Forward 5’-CTGCCACGGAGGTGTTATTACC-3’<br>Reverse 5’-GTGGCAGGTAAGATCGATCACCTC-3’	675
mRPL13a	Forward 5’-GCCTCTGCAGTTTGCTTA CC-3’<br>Reverse 5’- ATGTCAGAGCGGTTGCTT CT-3’	198

mRPL13a: mouse ribosomal protein large subunit 13a

Table S2. The gradient elution program for UPLC/MS-qTOF

Time(min)	Flow (mL/min)	Solution A (%)	Solution B (%)	Gradient Curve
0	0.4	99.9	0.1	–
2	0.4	99.9	0.1	6
7	0.4	44.0	56.0	6
8	0.4	44.0	56.0	6
8.01	0.4	99.9	0.1	6
9	0.4	99.9	0.1	6

A: 2% Acetonitrile/H2O + 0.1% Formic acid

B: 100%Acetonitrile + 0.1% Formic acid
